# {2-[(1,3-Benzo­thia­zol-2-yl)meth­oxy]-5-chloro­phen­yl}(4-chloro­phen­yl)methan­one

**DOI:** 10.1107/S1600536813016243

**Published:** 2013-06-19

**Authors:** K. N. Venugopala, Susanta K. Nayak, B. Odhav

**Affiliations:** a Department of Biotechnology and Food Technology, Durban University of Technology, Durban 4001, South Africa; b Equipe Chimie du Solide et Matériaux, UMR 6226 Institut des Sciences, Université de Rennes 1, Campus de Beaulieu, Avenue du Général Leclerc, 35042 Rennes cedex, France

## Abstract

In the title compound, C_21_H_13_Cl_2_NO_2_S, the benzo­thia­zole ring makes dihedral angles of 0.94 (1) and 70.65 (5)° with the 4-chloro­phenyl­methanone unit and the 5-chloro­phenyl ring, respectively. The dihedral angle between the 4-chloro­phenyl­methanone unit and the 5-chloro­phenyl ring is 66.20 (5)°. The crystal structure consists of dimeric units generated by C—H⋯N hydrogen bonds, further linked by C—H⋯O and C—H⋯π inter­actions, leading to a three-dimensional network.

## Related literature

For crystal structures of benzo­thia­zole derivatives, see: Venugopala *et al.* (2012 ▶); Nayak *et al.* (2013 ▶). For background to the applications of benzo­thia­zole derivatives, see: Rana *et al.* (2007 ▶); Saeed *et al.* (2010 ▶); Kelarev *et al.* (2003 ▶); Telvekar *et al.* (2012 ▶).
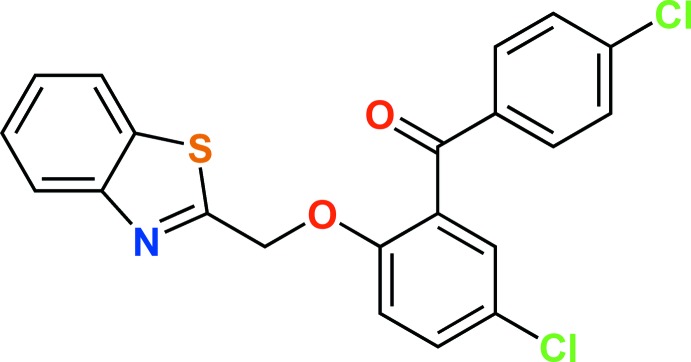


## Experimental

### 

#### Crystal data

C_21_H_13_Cl_2_NO_2_S
*M* *_r_* = 414.29
Monoclinic, 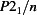

*a* = 13.6452 (4) Å
*b* = 7.47005 (19) Å
*c* = 18.7286 (6) Å
β = 105.772 (3)°
*V* = 1837.14 (9) Å^3^
*Z* = 4
Mo *K*α radiation
μ = 0.48 mm^−1^
*T* = 292 K
0.23 × 0.21 × 0.14 mm

#### Data collection

Oxford Diffraction Xcalibur (Eos, Nova) diffractometer
Absorption correction: multi-scan (*CrysAlis PRO*; Oxford Diffraction, 2010 ▶) *T*_min_ = 0.897, *T*_max_ = 0.935
18937 measured reflections
3602 independent reflections
2544 reflections with *I* > 2σ(*I*)
*R*_int_ = 0.047

#### Refinement

*R*[*F* ^2^ > 2σ(*F* ^2^)] = 0.043
*wR*(*F* ^2^) = 0.109
*S* = 1.08
3602 reflections
241 parameters
H-atom parameters constrained
Δρ_max_ = 0.24 e Å^−3^
Δρ_min_ = −0.29 e Å^−3^

### 

Data collection: *CrysAlis CCD* (Oxford Diffraction, 2009 ▶); cell refinement: *CrysAlis CCD*; data reduction: *CrysAlis RED* (Oxford Diffraction, 2009 ▶); program(s) used to solve structure: *SHELXS97* (Sheldrick, 2008 ▶); program(s) used to refine structure: *SHELXL97* (Sheldrick, 2008 ▶); molecular graphics: *ORTEP-3 for Windows* (Farrugia, 2012 ▶) and *Mercury* (Macrae *et al.*, 2008 ▶); software used to prepare material for publication: *PLATON* (Spek, 2009 ▶) and *PARST* (Nardelli, 1995 ▶).

## Supplementary Material

Crystal structure: contains datablock(s) global, I. DOI: 10.1107/S1600536813016243/lx2286sup1.cif

Structure factors: contains datablock(s) I. DOI: 10.1107/S1600536813016243/lx2286Isup2.hkl

Supplementary material file. DOI: 10.1107/S1600536813016243/lx2286Isup3.cml

Additional supplementary materials:  crystallographic information; 3D view; checkCIF report

## Figures and Tables

**Table 1:** Hydrogen-bond geometry (Å, °) *Cg* is the centroid of the S1/C1/C6/N1/C7 thia­zole ring.

*D*—H⋯*A*	*D*—H	H⋯*A*	*D*⋯*A*	*D*—H⋯*A*
C5—H5⋯O2^i^	0.93	2.56	3.429 (3)	157
C17—H17⋯N1^ii^	0.93	2.62	3.442 (3)	148
C18—H18⋯*Cg* ^iii^	0.93	2.83	3.682 (2)	152

Symmetry codes: (i) 
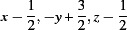
; (ii) 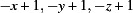; (iii) 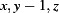.

**Fig. 1.**
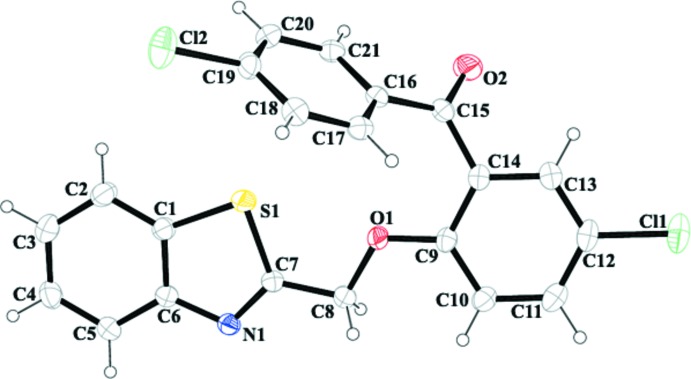
The molecular structure of the title compound with the atom numbering scheme. Displacement ellipsoids are drawn at the 30% probability level. H atoms are presented as small spheres of arbitrary radius.

**Fig. 2.**
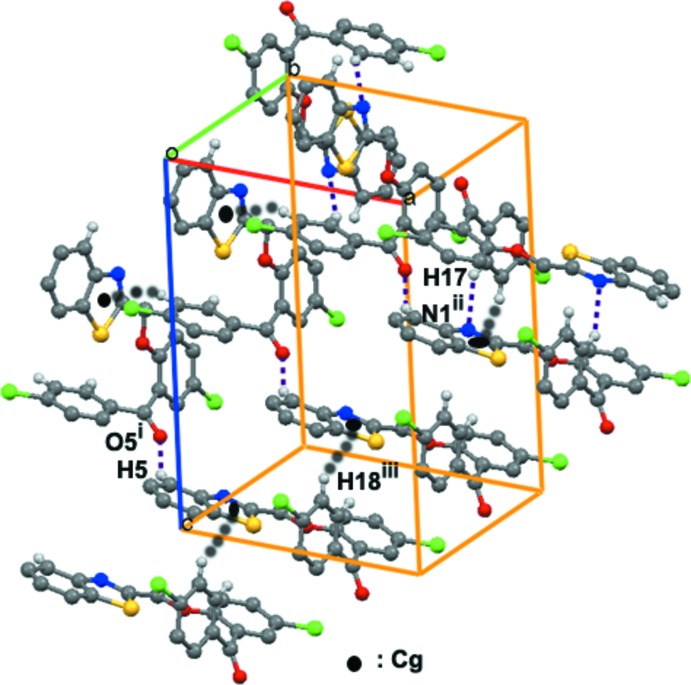
A view of the C–H···N, C–H..O and C–H···π interactions (dotted lines) in the crystal structure of the title compound. H atoms non-participating in hydrogen-bonding were omitted for clarity. [Symmetry codes: (i)x-1/2,-y+3/2,z-1/2; (ii)-x+1,-y+1,-z+1; (iii)x,y-1, z]

## supplementary crystallographic information

### Comment

Substituted benzothiazole derivatives have been reported to exhibit various pharmacological properties such as analgesic, antibacterial, antifungal, antidepressant, antitumor, antihypertensive, anthelmintic, and herbicidal activity (Kelarev *et al.* (2003)). However, the variety of biological and structural features of new benzothiazole derivatives is of great scientific interest (Rana *et al.* (2007); Telvekar *et al.* (2012); Saeed *et al.* (2010) and Nayak *et al.* (2013)). In continuation of our interest on such molecules (Venugopala *et al.* (2012)) here, we report the single-crystal structure of the title compound.

In the title molecule (Fig. 1), the benzothiazole ring makes dihedral angles of 0.94 (1)° and 70.65 (5)° with the 4-chlorophenylmethanone unit and the 5-chlorophenyl ring, respectively. The dihedral angle between the 4-chlorophenylmethanone unit and the 5-chlorophenyl ring is 66.20 (5)°. The crystal structure consists of dimeric units generated by C–H···N hydrogen bonds, further linked by C–H···O and C–H···π interactions, which lead to a three-dimensional network (Table 1 and Fig. 2, Cg is the centroid of the S1/C1/C6/N1/C7 thiazole ring). The crystal structure consists of dimeric units generated by C–H···N hydrogen bonds, further linked by C–H···O and C–H···π interactions, which lead to a three-dimensional network (Table 1 and Fig. 2).

### Experimental

A mixture of (2-chloromethyl)benzo[*d*]thiazole (1 mmol) and (5-chloro-2-hydroxyphenyl)(4-chlorophenyl)methanone (1 mmol) and in dry THF, dry potassium carbonate (1 mmol) was added and stirred at room temperature. The reaction mixture was added and the reaction mixture was stirred at room temperature for 14 h. The reaction mixture was concentrated to remove the solvent, diluted with ethyl acetate, washed with water, brine solution and dried over anhydrous sodium sulfate. The organic layer was concentrated to yield a residue which was purified by column chromatography using ethyl acetate and n-hexane as eluent (7:3, Rf = 0.73) to afford the product in 83% as a brown solid (m. p. 448 (2) K). Suitable crystals for single-crystal X-ray study were obtained from dichloromethane solvent using slow evaporation technique at room temperature.

### Refinement

All H atoms were positioned geometrically and refined using a riding model, with C–H = 0.95 Å for aryl and 0.99 Å for methylene H atoms. *U*_iso_(H) = 1.2*U*_eq_(C) for aryl and methylene H atoms.

### Crystal data

**Table:**

C_21_H_13_Cl_2_NO_2_S	*F*(000) = 848
*M**_r_* = 414.29	*D*_x_ = 1.498 Mg m^−^^3^
Monoclinic, *P*2_1_/*n*	Melting point: 448(2) K
Hall symbol: -P 2yn	Mo *K*α radiation, λ = 0.7107 Å
*a* = 13.6452 (4) Å	Cell parameters from 340 reflections
*b* = 7.47005 (19) Å	θ = 1.0–28.0°
*c* = 18.7286 (6) Å	µ = 0.48 mm^−^^1^
β = 105.772 (3)°	*T* = 292 K
*V* = 1837.14 (9) Å^3^	Plate, colourless
*Z* = 4	0.23 × 0.21 × 0.14 mm

### Data collection

**Table:**

Oxford Diffraction Xcalibur (Eos, Nova) diffractometer	3602 independent reflections
Radiation source: Mova (Mo) X-ray Source	2544 reflections with *I* > 2σ(*I*)
Mirror monochromator	*R*_int_ = 0.047
Detector resolution: 16.0839 pixels mm^-1^	θ_max_ = 26.0°, θ_min_ = 3.0°
ω scans	*h* = −16→16
Absorption correction: multi-scan (*CrysAlis PRO*; Oxford Diffraction, 2010)	*k* = −9→9
*T*_min_ = 0.897, *T*_max_ = 0.935	*l* = −23→22
18937 measured reflections	

### Refinement

**Table:**

Refinement on *F*^2^	Primary atom site location: structure-invariant direct methods
Least-squares matrix: full	Secondary atom site location: difference Fourier map
*R*[*F*^2^ > 2σ(*F*^2^)] = 0.043	Hydrogen site location: difference Fourier map
*wR*(*F*^2^) = 0.109	H-atom parameters constrained
*S* = 1.08	*w* = 1/[σ^2^(*F*_o_^2^) + (0.0429*P*)^2^ + 0.3056*P*] where *P* = (*F*_o_^2^ + 2*F*_c_^2^)/3
3602 reflections	(Δ/σ)_max_ = 0.001
241 parameters	Δρ_max_ = 0.24 e Å^−^^3^
0 restraints	Δρ_min_ = −0.29 e Å^−^^3^

### Special details

**Table:**

Geometry. All s.u.'s (except the s.u. in the dihedral angle between two l.s. planes) are estimated using the full covariance matrix. The cell s.u.'s are taken into account individually in the estimation of s.u.'s in distances, angles and torsion angles; correlations between s.u.'s in cell parameters are only used when they are defined by crystal symmetry. An approximate (isotropic) treatment of cell s.u.'s is used for estimating s.u.'s involving l.s. planes.
Refinement. Refinement of F^2^ against ALL reflections. The weighted R-factor wR and goodness of fit S are based on F^2^, conventional R-factors R are based on F, with F set to zero for negative F^2^. The threshold expression of F^2^ > 2σ(F^2^) is used only for calculating R-factors(gt) etc. and is not relevant to the choice of reflections for refinement. R-factors based on F^2^ are statistically about twice as large as those based on F, and R- factors based on ALL data will be even larger.

### Fractional atomic coordinates and isotropic or equivalent isotropic displacement parameters (Å^2^)

**Table:**

	*x*	*y*	*z*	*U*_iso_*/*U*_eq_	
Cl1	1.03821 (5)	0.35913 (10)	0.66313 (5)	0.0683 (2)	
Cl2	0.27764 (6)	0.15904 (12)	0.68234 (5)	0.0776 (3)	
S1	0.41607 (5)	0.64525 (9)	0.62033 (3)	0.0465 (2)	
O1	0.61507 (12)	0.6008 (2)	0.61055 (9)	0.0449 (4)	
O2	0.75637 (13)	0.4439 (3)	0.80903 (9)	0.0571 (5)	
N1	0.39772 (14)	0.8383 (2)	0.50261 (9)	0.0357 (4)	
C1	0.29751 (17)	0.7360 (3)	0.57862 (12)	0.0384 (5)	
C2	0.20652 (19)	0.7169 (3)	0.59779 (13)	0.0496 (7)	
H2	0.2033	0.6494	0.6388	0.060*	
C3	0.12177 (14)	0.8006 (3)	0.55448 (11)	0.0551 (7)	
H3	0.0600	0.7892	0.5661	0.066*	
C4	0.12656 (14)	0.9025 (3)	0.49327 (11)	0.0524 (7)	
H4	0.0681	0.9592	0.4652	0.063*	
C5	0.21558 (18)	0.9210 (3)	0.47357 (12)	0.0433 (6)	
H5	0.2179	0.9891	0.4325	0.052*	
C6	0.30286 (17)	0.8354 (3)	0.51649 (11)	0.0344 (5)	
C7	0.46196 (17)	0.7448 (3)	0.55169 (11)	0.0335 (5)	
C8	0.56983 (17)	0.7178 (3)	0.55111 (12)	0.0410 (6)	
H8A	0.5729	0.6658	0.5043	0.049*	
H8B	0.6056	0.8315	0.5574	0.049*	
C9	0.71332 (16)	0.5456 (3)	0.61952 (12)	0.0359 (5)	
C10	0.76869 (18)	0.5827 (3)	0.56916 (13)	0.0423 (6)	
H10	0.7387	0.6468	0.5262	0.051*	
C11	0.86790 (19)	0.5250 (3)	0.58258 (14)	0.0476 (6)	
H11	0.9046	0.5490	0.5485	0.057*	
C12	0.91267 (17)	0.4315 (3)	0.64669 (15)	0.0455 (6)	
C13	0.85941 (17)	0.3962 (3)	0.69754 (13)	0.0406 (6)	
H13	0.8910	0.3356	0.7411	0.049*	
C14	0.75846 (16)	0.4504 (3)	0.68441 (12)	0.0352 (5)	
C15	0.70758 (17)	0.4179 (3)	0.74501 (13)	0.0364 (5)	
C16	0.60070 (17)	0.3519 (3)	0.72806 (11)	0.0328 (5)	
C17	0.55910 (17)	0.2434 (3)	0.66717 (12)	0.0375 (5)	
H17	0.5975	0.2137	0.6348	0.045*	
C18	0.46132 (18)	0.1796 (3)	0.65457 (13)	0.0413 (6)	
H18	0.4343	0.1034	0.6148	0.050*	
C19	0.40357 (17)	0.2297 (3)	0.70148 (13)	0.0426 (6)	
C20	0.44320 (18)	0.3374 (3)	0.76184 (13)	0.0435 (6)	
H20	0.4035	0.3705	0.7929	0.052*	
C21	0.54231 (18)	0.3957 (3)	0.77580 (12)	0.0388 (6)	
H21	0.5704	0.4650	0.8176	0.047*	

### Atomic displacement parameters (Å^2^)

**Table:**

	*U*^11^	*U*^22^	*U*^33^	*U*^12^	*U*^13^	*U*^23^
Cl1	0.0348 (4)	0.0681 (5)	0.1045 (6)	0.0057 (3)	0.0233 (4)	−0.0048 (4)
Cl2	0.0439 (5)	0.0873 (6)	0.1010 (6)	−0.0192 (4)	0.0186 (4)	−0.0089 (5)
S1	0.0465 (4)	0.0513 (4)	0.0448 (4)	0.0111 (3)	0.0174 (3)	0.0183 (3)
O1	0.0344 (9)	0.0520 (10)	0.0520 (10)	0.0117 (8)	0.0180 (8)	0.0215 (8)
O2	0.0495 (11)	0.0790 (14)	0.0378 (10)	−0.0129 (10)	0.0032 (9)	−0.0004 (9)
N1	0.0373 (11)	0.0370 (11)	0.0321 (10)	0.0027 (8)	0.0084 (9)	0.0035 (8)
C1	0.0397 (14)	0.0370 (13)	0.0398 (13)	0.0046 (10)	0.0131 (11)	0.0034 (10)
C2	0.0507 (17)	0.0545 (16)	0.0502 (15)	0.0031 (13)	0.0245 (13)	0.0062 (13)
C3	0.0427 (16)	0.0652 (18)	0.0626 (18)	0.0036 (13)	0.0231 (14)	0.0006 (14)
C4	0.0386 (15)	0.0629 (18)	0.0525 (16)	0.0123 (13)	0.0068 (13)	0.0026 (13)
C5	0.0421 (15)	0.0465 (14)	0.0390 (14)	0.0040 (11)	0.0068 (12)	0.0054 (11)
C6	0.0379 (14)	0.0325 (12)	0.0327 (12)	−0.0002 (10)	0.0093 (11)	−0.0035 (10)
C7	0.0366 (13)	0.0302 (12)	0.0340 (12)	0.0004 (10)	0.0102 (10)	0.0004 (10)
C8	0.0385 (14)	0.0431 (14)	0.0422 (14)	0.0059 (11)	0.0123 (11)	0.0114 (11)
C9	0.0321 (13)	0.0318 (12)	0.0454 (14)	0.0011 (10)	0.0131 (11)	−0.0007 (10)
C10	0.0433 (15)	0.0406 (14)	0.0468 (14)	−0.0005 (11)	0.0187 (12)	0.0018 (11)
C11	0.0454 (15)	0.0439 (15)	0.0608 (16)	−0.0051 (12)	0.0270 (13)	−0.0046 (13)
C12	0.0309 (13)	0.0403 (14)	0.0673 (17)	−0.0023 (11)	0.0166 (13)	−0.0105 (13)
C13	0.0348 (14)	0.0359 (13)	0.0487 (14)	0.0006 (10)	0.0073 (12)	−0.0020 (11)
C14	0.0320 (13)	0.0300 (12)	0.0433 (13)	−0.0014 (10)	0.0100 (11)	−0.0038 (10)
C15	0.0376 (14)	0.0318 (12)	0.0377 (14)	0.0040 (10)	0.0066 (11)	0.0034 (10)
C16	0.0354 (13)	0.0303 (12)	0.0313 (12)	0.0020 (10)	0.0065 (10)	0.0043 (9)
C17	0.0453 (15)	0.0316 (13)	0.0371 (13)	0.0047 (11)	0.0136 (11)	0.0012 (10)
C18	0.0437 (15)	0.0339 (13)	0.0440 (14)	−0.0028 (11)	0.0078 (12)	−0.0039 (10)
C19	0.0327 (13)	0.0417 (14)	0.0516 (15)	−0.0038 (11)	0.0083 (12)	0.0077 (12)
C20	0.0440 (15)	0.0500 (15)	0.0406 (14)	0.0002 (12)	0.0184 (12)	0.0038 (11)
C21	0.0452 (15)	0.0412 (14)	0.0295 (12)	−0.0018 (11)	0.0094 (11)	−0.0014 (10)

### Geometric parameters (Å, º)

**Table:**

Cl1—C12	1.742 (2)	C9—C10	1.387 (3)
Cl2—C19	1.739 (2)	C9—C14	1.398 (3)
S1—C1	1.732 (2)	C10—C11	1.377 (3)
S1—C7	1.742 (2)	C10—H10	0.9300
O1—C9	1.369 (2)	C11—C12	1.380 (3)
O1—C8	1.418 (3)	C11—H11	0.9300
O2—C15	1.218 (3)	C12—C13	1.372 (3)
N1—C7	1.290 (3)	C13—C14	1.392 (3)
N1—C6	1.388 (3)	C13—H13	0.9300
C1—C2	1.390 (3)	C14—C15	1.502 (3)
C1—C6	1.399 (3)	C15—C16	1.490 (3)
C2—C3	1.370 (3)	C16—C17	1.389 (3)
C2—H2	0.9300	C16—C21	1.389 (3)
C3—C4	1.3923	C17—C18	1.375 (3)
C3—H3	0.9300	C17—H17	0.9300
C4—C5	1.369 (3)	C18—C19	1.382 (3)
C4—H4	0.9300	C18—H18	0.9300
C5—C6	1.398 (3)	C19—C20	1.373 (3)
C5—H5	0.9300	C20—C21	1.376 (3)
C7—C8	1.489 (3)	C20—H20	0.9300
C8—H8A	0.9700	C21—H21	0.9300
C8—H8B	0.9700		
			
C1—S1—C7	88.81 (11)	C9—C10—H10	119.9
C9—O1—C8	119.01 (16)	C10—C11—C12	119.9 (2)
C7—N1—C6	110.21 (18)	C10—C11—H11	120.1
C2—C1—C6	121.4 (2)	C12—C11—H11	120.1
C2—C1—S1	129.33 (18)	C13—C12—C11	120.6 (2)
C6—C1—S1	109.22 (17)	C13—C12—Cl1	119.9 (2)
C3—C2—C1	118.0 (2)	C11—C12—Cl1	119.50 (19)
C3—C2—H2	121.0	C12—C13—C14	120.5 (2)
C1—C2—H2	121.0	C12—C13—H13	119.8
C2—C3—C4	121.07 (13)	C14—C13—H13	119.8
C2—C3—H3	119.5	C13—C14—C9	118.9 (2)
C4—C3—H3	119.5	C13—C14—C15	117.2 (2)
C5—C4—C3	121.37 (13)	C9—C14—C15	123.62 (19)
C5—C4—H4	119.3	O2—C15—C16	120.2 (2)
C3—C4—H4	119.3	O2—C15—C14	118.5 (2)
C4—C5—C6	118.6 (2)	C16—C15—C14	121.3 (2)
C4—C5—H5	120.7	C17—C16—C21	119.2 (2)
C6—C5—H5	120.7	C17—C16—C15	121.8 (2)
N1—C6—C5	125.3 (2)	C21—C16—C15	119.0 (2)
N1—C6—C1	115.25 (19)	C18—C17—C16	120.3 (2)
C5—C6—C1	119.5 (2)	C18—C17—H17	119.9
N1—C7—C8	123.36 (19)	C16—C17—H17	119.9
N1—C7—S1	116.50 (17)	C17—C18—C19	119.4 (2)
C8—C7—S1	120.13 (16)	C17—C18—H18	120.3
O1—C8—C7	107.34 (17)	C19—C18—H18	120.3
O1—C8—H8A	110.2	C20—C19—C18	121.1 (2)
C7—C8—H8A	110.2	C20—C19—Cl2	119.58 (19)
O1—C8—H8B	110.2	C18—C19—Cl2	119.27 (19)
C7—C8—H8B	110.2	C19—C20—C21	119.3 (2)
H8A—C8—H8B	108.5	C19—C20—H20	120.4
O1—C9—C10	123.5 (2)	C21—C20—H20	120.4
O1—C9—C14	116.45 (18)	C20—C21—C16	120.6 (2)
C10—C9—C14	120.0 (2)	C20—C21—H21	119.7
C11—C10—C9	120.2 (2)	C16—C21—H21	119.7
C11—C10—H10	119.9		
			
C7—S1—C1—C2	177.6 (2)	C10—C11—C12—Cl1	−179.97 (18)
C7—S1—C1—C6	−0.48 (17)	C11—C12—C13—C14	1.6 (3)
C6—C1—C2—C3	−0.9 (4)	Cl1—C12—C13—C14	−178.83 (17)
S1—C1—C2—C3	−178.70 (18)	C12—C13—C14—C9	−1.7 (3)
C1—C2—C3—C4	−0.3 (3)	C12—C13—C14—C15	−175.9 (2)
C2—C3—C4—C5	0.85 (17)	O1—C9—C14—C13	−177.90 (19)
C3—C4—C5—C6	−0.2 (3)	C10—C9—C14—C13	0.7 (3)
C7—N1—C6—C5	−179.2 (2)	O1—C9—C14—C15	−4.1 (3)
C7—N1—C6—C1	0.3 (3)	C10—C9—C14—C15	174.5 (2)
C4—C5—C6—N1	178.5 (2)	C13—C14—C15—O2	40.4 (3)
C4—C5—C6—C1	−0.9 (3)	C9—C14—C15—O2	−133.5 (2)
C2—C1—C6—N1	−178.0 (2)	C13—C14—C15—C16	−138.4 (2)
S1—C1—C6—N1	0.2 (2)	C9—C14—C15—C16	47.7 (3)
C2—C1—C6—C5	1.5 (3)	O2—C15—C16—C17	−148.8 (2)
S1—C1—C6—C5	179.73 (18)	C14—C15—C16—C17	30.0 (3)
C6—N1—C7—C8	178.5 (2)	O2—C15—C16—C21	29.2 (3)
C6—N1—C7—S1	−0.7 (2)	C14—C15—C16—C21	−152.1 (2)
C1—S1—C7—N1	0.70 (18)	C21—C16—C17—C18	−0.2 (3)
C1—S1—C7—C8	−178.48 (19)	C15—C16—C17—C18	177.7 (2)
C9—O1—C8—C7	176.11 (18)	C16—C17—C18—C19	2.3 (3)
N1—C7—C8—O1	−176.87 (19)	C17—C18—C19—C20	−2.1 (3)
S1—C7—C8—O1	2.3 (3)	C17—C18—C19—Cl2	176.40 (17)
C8—O1—C9—C10	−6.9 (3)	C18—C19—C20—C21	−0.2 (4)
C8—O1—C9—C14	171.7 (2)	Cl2—C19—C20—C21	−178.73 (18)
O1—C9—C10—C11	179.0 (2)	C19—C20—C21—C16	2.3 (3)
C14—C9—C10—C11	0.4 (3)	C17—C16—C21—C20	−2.1 (3)
C9—C10—C11—C12	−0.6 (4)	C15—C16—C21—C20	179.9 (2)
C10—C11—C12—C13	−0.4 (4)		

### Hydrogen-bond geometry (Å, º)

Cg is the centroid of the S1/C1/C6/N1/C7 thiazole ring.

**Table:**

*D*—H···*A*	*D*—H	H···*A*	*D*···*A*	*D*—H···*A*
C5—H5···O2^i^	0.93	2.56	3.429 (3)	157
C17—H17···N1^ii^	0.93	2.62	3.442 (3)	148
C18—H18···*Cg*^iii^	0.93	2.83	3.682 (2)	152

Symmetry codes: (i) *x*−1/2, −*y*+3/2, *z*−1/2; (ii) −*x*+1, −*y*+1, −*z*+1; (iii) *x*, *y*−1, *z*.
